# Replication of G Quadruplex DNA

**DOI:** 10.3390/genes10020095

**Published:** 2019-01-29

**Authors:** Leticia Koch Lerner, Julian E. Sale

**Affiliations:** MRC Laboratory of Molecular Biology, Francis Crick Avenue, Cambridge CB2 0QH, UK; leticia@mrc-lmb.cam.ac.uk

**Keywords:** G quadruplex, DNA replication, DNA helicases, DNA secondary structure

## Abstract

A cursory look at any textbook image of DNA replication might suggest that the complex machine that is the replisome runs smoothly along the chromosomal DNA. However, many DNA sequences can adopt non-B form secondary structures and these have the potential to impede progression of the replisome. A picture is emerging in which the maintenance of processive DNA replication requires the action of a significant number of additional proteins beyond the core replisome to resolve secondary structures in the DNA template. By ensuring that DNA synthesis remains closely coupled to DNA unwinding by the replicative helicase, these factors prevent impediments to the replisome from causing genetic and epigenetic instability. This review considers the circumstances in which DNA forms secondary structures, the potential responses of the eukaryotic replisome to these impediments in the light of recent advances in our understanding of its structure and operation and the mechanisms cells deploy to remove secondary structure from the DNA. To illustrate the principles involved, we focus on one of the best understood DNA secondary structures, G quadruplexes (G4s), and on the helicases that promote their resolution.

## 1. The Nature of DNA Secondary Structures

Although the majority of DNA within a cell exists in the canonical double-stranded B-form, transactions on DNA such as transcription and replication require the duplex to be unwound. When single stranded, DNA has the opportunity to fold into a variety of non-B conformations, some of which can be more stable than double-stranded DNA itself. In this review, we will refer generically to these non-B conformations as secondary structures. The primary determinant of the form of the structure is the DNA sequence. Secondary structure formation is generally a feature of a low-complexity or repetitive sequence, but is influenced by factors such as symmetry and the availability of additional nucleic acid strands, for instance RNA. The simplest DNA secondary structures formed are hairpins and cruciforms, which tend to arise in inverted and mirror repeat sequences. Many tandem repeats can also adopt secondary structures: for instance, polypurine tracts can form triplex or hinged-DNA (H-DNA) while repetitive G-rich sequences may form G quadruplexes (G4s) [1,2]. In this review we will focus on G4s. However, in the context of DNA replication, many of the general principles discussed can be applied to the broad range of structural impediments.

**Figure 1:**
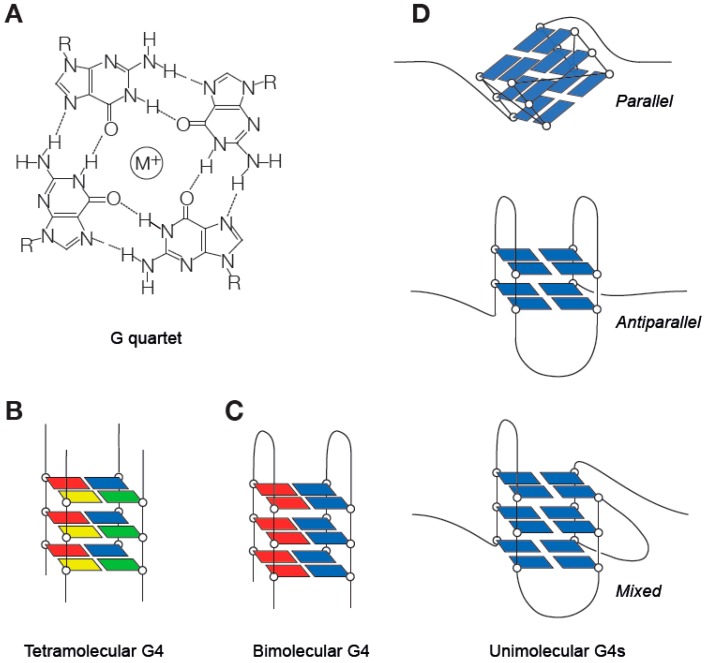
G quartets and G quadruplexes. (**A**) A G quartet. Four G bases form a planar tetrad stabilised by Hoogsteen bonding and a central monovalent metal ion. (**B**–**D**) Basic forms of the G quadruplex. (**B**) A tetramolecular G4. (**C**) A bimolecular G4. (**D**) Three conformations of unimolecular G4 with different backbone arrangements, parallel, anti-parallel and mixed.

G4 DNA secondary structures arise because of the ability of guanine to Hoogsteen base pair with itself to create ring-like formations known as G quartets (Figure 1) [3]. G quartets can stack through π–π interactions to form G4s (Figure 1) [4,5,6]. G4s can form from four strands of DNA (a tetramolecular G4, a form frequently studied in vitro but with questionable in vivo significance), two strands (a bimolecular G4, which can link two DNA strands together) and, most relevant in the context of DNA replication, a unimolecular G4 formed by folding of a single strand of DNA (Figure 1). Initial biophysical studies led to the definition of a consensus G4 motif of G_3–5_N_1–7_G_3–5_N_1–7_G_3–5_N_1–7_G_3–5_, in which N can be any nucleotide [7,8]. However, more recent work has demonstrated that a considerably wider range of sequences, for instance with G runs of 2 or with N being significantly greater than 7, can form G4s and exert biological effects [9,10]. Consistent with this, a genome-wide sequencing-based assay suggests that there are more than 700,000 sites with G4-forming potential in the human genome [11]. Intramolecular G4 structures exhibit considerable topological diversity, a few common examples of which are shown in Figure 1D. This diversity is largely dependent upon the precise sequence of the G4 motif, which determines the maximum height of the G quartet stack and length of the loops formed by bases not participating in the quartets [12].

While G4 formation has been studied in vitro for many years, it is only recently that converging lines of evidence have led to widespread acceptance of their existence and impact in vivo [13,14,15,16]. While not the focus of this review, it is worth noting that the physiological functions of G4s remain a topic of debate. They have been linked to transcriptional regulation [17], telomere maintenance [18,19] and replication origin specification [20,21,22], to give just a few examples, but in general, their biological role remains poorly understood. Indeed, it is perfectly possible that many of the sequences that are capable of forming G4s do not have a specific function. Nonetheless, since G4s can act as potent blocks to DNA synthesis in vitro [23] and in vivo [24], all of these sites pose a potential challenge to DNA replication. Indeed, G4s are associated with an increased risk of potentially deleterious genetic and epigenetic events linked to replication [24,25,26,27].

## 2. A Technical Aside: Monitoring the Response of the Replisome to Sites of Secondary Structure Formation In Vivo

Critical to understanding the interaction between the formation of DNA secondary structure and replication are sensitive assays that monitor the response of forks to impediments. A number of approaches have been adopted to this problem over the years. Physical monitoring of replication forks encountering genomic secondary structures is challenging because fork stalling at these sites is often transient. In bacteria and yeast, the use of 2-dimensional gel electrophoresis to monitor replication of a specific restriction fragment containing a secondary structure has been very informative, but is relatively insensitive as it requires a substantial number of forks to stall in order for a signal to be detected [28,29]. In vertebrate cells this technique is even more challenging when applied to genomic sequences. However, it has been used very effectively in replicating plasmids [30] and the approach can be further enhanced by combining this with electron microscopy of the replication intermediates, an approach that has been used to demonstrate fork-reversal at (GAA)*_n_* repeats [31]. However, SV40 plasmid replication is unlikely to perfectly recapitulate the supercoiling, chromatin structure and replication of chromosomal DNA. Direct monitoring of fork progression using DNA combing requires knowledge of the location of potential structure-forming sequences, which means using fluorescent in situ hybridisation (FISH) on single DNA fibres. This has been achieved at telomeres [32] and at the *FXN* and *FMR1* loci in human cells [33,34], but is enormously time-consuming and of limited sensitivity. Recently, an elegant in vivo method that determines fork progression between two points in the yeast genome by monitoring the doubling of intensity of adjacent *lacI* and *tetR* arrays bound with fluorescent proteins has yielded promising results [35].

Monitoring genetic instability of loci containing structure-forming sequences, either by use of a reporter assay [36,37,38] or directly using methods like Southern blotting [39] has also been very informative. Since the genetic changes are propagated through the population, their frequency can be estimated using a classical fluctuation analysis [40]. More recently, genome-wide deep sequencing approaches have identified sequences that are intrinsically prone to drive replication fork stress and breakage [41,42]. While genetic changes at structure-forming DNA are relatively easy to monitor in an expanding cell population, it is likely that such events significantly underestimate the frequency of fork pausing. We have shown in chicken DT40 cells that replication fork pausing at DNA secondary structures can produce local and heritable epigenetic changes that are propagated through an expanding population and can thus be used to indirectly derive a rate of fork pausing by fluctuation analysis [9,24]. We have proposed that this phenomenon results from local uncoupling of the DNA helicase from the replicative polymerase, which in turn uncouples DNA synthesis from the usually tight coupling of histone recycling and fork progression [43]. This results in localised loss of epigenetic information in the vicinity of the secondary structure which, crucially, in this system, leads to a stable and heritable change in gene expression that is propagated through the cell population. The *BU-1* locus of DT40, which encodes a surface glycoprotein, provides a tractable physical manifestation of this phenomenon [9]. Stochastic G4-dependent instability of *BU-1* expression is readily monitored and, uniquely, provides a cumulative and sensitive record of replisome stalling at a specific G4 in vivo [9,44].

G4s provide a convenient paradigm for thinking about the response of the replisome to secondary structures. Although it is not currently possible to be absolutely certain of the precise conformation adopted by a given G4 in vivo, the ability to control the potential for secondary structure formation with defined point mutations allows strong correlations to be drawn between in vitro behaviour and in vivo effects.

## 3. G Quadruplex (G4)-Forming Potential in the Genome Is Associated with Genetic and Epigenetic Instability

Extensive evidence from a wide range of organisms has correlated sites of potential DNA secondary structure formation with mutagenesis. Mechanistically, it is thought that structures can induce mutagenesis both as a result of their intrinsic capacity to impede DNA replication [45] and from their ability to modulate the mutability of DNA by exogenous agents [46,47]. There is a clear link between DNA secondary structure formation and many of the hallmark features of cancer genomes, including translocation breakpoints, indels, copy number variation and point mutagenesis [48,49,50]. Again, the ability of structures to impede DNA replication is central to the generation of this genetic instability [26]. More recently, sites of potential secondary structure formation have been linked to epigenetic changes [24,25], which are also a potentially fertile source of diversity upon which selection could act during the evolution of cancer [51]. Therefore, it is crucial to establish a detailed understanding of how DNA replication forks interact with DNA secondary structures.

## 4. Evidence That G4s Can Interfere with Both Lagging and Leading Strand Replication

A persistent question has been whether secondary structures are more likely to form on the leading or lagging strand during DNA replication. The two possibilities are not mutually exclusive but, given the intrinsic asymmetry of DNA replication, there are specific considerations in each case.

### 4.1. Lagging Strand G4s

The discontinuous nature of lagging strand replication means that the lagging strand template will remain single-stranded for longer than the leading strand. This led many early models for replication-associated G4 formation to suggest the lagging strand template will be intrinsically more prone to G4 formation [52,53]. However, the unwound lagging strand is thought to become coated with the single strand binding protein RPA, which is effective at countering G4s [54]. A prominent lagging strand G4-forming sequence is seen in telomeres, which in vertebrates comprise the repeated sequence TTAGGG, and fork progression through the telomeric repeats is slow and prone to instability [32]. Furthermore, very recent work has shown how replication in yeast is delayed by lagging strand G4s in the absence of the PIF1 helicase [55]. These data are consistent with the formation of lagging strand G4s, but the reason for replication delay given the continuous ability to reprime this strand remains unclear.

### 4.2. Leading Strand G4s

There is now strong in vivo evidence that G4s can also cause problems on the leading strand template, particularly in the absence of specialised helicases and other factors that promote G4 unwinding. In budding yeast, the G4-forming human minisatellite CEB1 is well tolerated on a leading strand template but exhibits significant genetic instability when cells are treated with a G4-stabilising ligand or when the helicase PIF1 is disrupted [56]. Using our approach of monitoring epigenetic instability in DT40 cells, we have shown that G4s can interrupt leading strand DNA synthesis when cells lack a number of factors e.g. the specialised polymerase REV1, specialised helicases FANCJ, BLM and WRN [9,24,57], and when G4s are stabilised with ligands in wild-type cells [44,58]. Importantly, we have also shown that the effects of a leading strand template G4 on epigenetic stability can be mitigated by repriming by the primase/polymerase PrimPol [9]. Strikingly, the absence of PrimPol, which does not itself directly contribute to the replication of G4s in vitro [59], results in an extremely high per division probability of *BU-1* expression instability, approaching 0.1. This suggests strongly that G4 formation does indeed frequently impede the leading strand polymerases even though there is no mutagenic outcome [59].

Having established that G4s can impact replication with knock-on effects on genetic and epigenetic stability, in the next section we consider the factors that might influence the transition from duplex to quadruplex DNA in vivo.

## 5. When Do G4s Form and How Do They Interact with the Replisome?

What conditions favour transition of B-form DNA to a secondary structure? Clearly, DNA sequence is an important determinant with GC-rich regions naturally containing more potential G4 motifs. However, in addition, GC-rich domains disfavour nucleosomes [60] and nucleosome depletion could favour G4 formation [61]. If nucleosomes do indeed counter G4s, ensuring full nucleosome occupancy may be important in preventing genetic instability in G4-prone sequences. A potential example of this is seen in the action of the chromatin remodeller ATRX at telomeres. ATRX is able to bind to G4s [62] and through interaction with the histone chaperone DAXX is able to introduce the variant histone H3.3 into telomeres [63], a reaction that helps restrict inter-telomeric recombination, possibly as a consequence of reducing G4 formation [64].

Within sequences with structure-forming potential, superhelical stress per se may able to drive structural transitions, for instance the formation of H-DNA in homopurine:homopyrimidine mirror repeats [65]. However, this may not apply to all secondary structures, including G4s [66]. A more potent and reliable promoter of G4 formation is duplex unwinding during replication and transcription, which generates single-stranded DNA, which in turn favours G4 formation [67]. What is the minimum length of single-stranded DNA that needs to be exposed to allow G4 formation? Biophysically, this is reasonably straightforward to answer: a thermodynamically stable G4 can be formed in an appropriate sequence, e.g., (G_3_N_1_)_3_G_3_ [68], which is just 15 bp. However, in vivo, other factors need to be considered, not least of which is the state of the adjacent DNA, the torsional and longitudinal forces locally acting on the DNA and the presence of DNA-binding proteins and nucleosomes.

### 5.1. How Could Transcription Promote G4 Formation?

Transcription by RNA polymerase requires exposure of single-stranded DNA. However, the single-stranded bubble generated by RNAPII is likely to be quite short, in the order of eight nucleotides [69]. It is also well protected by the RNAPII complex and thus not amenable to G4 formation. However, the transcription bubble can become extended when RNAPII pauses [70] and this could potentially lead to the exposure of sufficient single-stranded DNA to allow the formation of G4s. Indeed, under these circumstances, the separation of the DNA strands allows the nascent RNA to hybridise to the template DNA preventing the coding strand of DNA reannealing. This three-stranded structure is called an R-loop [71] and in DNA sequences with structure-forming potential R-loop formation can facilitate or stabilise secondary structure formation in the exposed single DNA strand [72,73,74].

In addition to the single-stranded DNA exposed around the RNA polymerase, transcription also generates negatively supercoiled DNA in the wake of the elongating polymerase. In highly transcribed genes, the rate of supercoiling can exceed the ability of topoisomerases to counteract it resulting in a region of constitutive net negative helicity [75]. In turn, this negative supercoiling leads to transient denaturation of the DNA to form transient single-stranded ‘bubbles’ [76]. These transcription-associated single-stranded bubbles have been proposed to promote mutagenesis by activation-induced deaminase (AID) and they promote R-loop formation [77,78]. Therefore, it is conceivable that they also drive secondary structure formation within the DNA. While this idea has been confirmed biophysically for a number of secondary structures, including hairpins and cruciforms [79,80,81] and triplex DNA [82,83,84], G4s do not seem to form spontaneously in negatively supercoiled plasmids [66]. Nonetheless, G4 formation has been proposed as a possible sink for negative torsional stress generated during transcription in the absence of topoisomerase I [85].

### 5.2. G4 Formation and the Replisome.

Although there is clear evidence that G4s are able to impede the progression of the replisome, there remain many unanswered questions. When do G4s that interfere with the replisome form? Is formation more likely on the leading or lagging strand and, given the intrinsic asymmetry of the replication fork, are they handled differently on the two strands? What are the mechanisms by which replication-blocking G4s are resolved? In the next sections, we will attempt to address some of these issues in the context of our current understanding of the structure and mechanism of the eukaryotic replisome.

### 5.3. Pre-Formed G4s Encountered by the Replisome

Antibodies raised against G4s detect signals in non-S phase cells [86] suggesting that preformed G4s could be encountered by the replisome. In such encounters the structure will first meet the replicative CMG helicase, which unwinds the DNA duplex to create the single-stranded template for leading and lagging strand synthesis. To understand how preformed G4s might be dealt with by replisome, it is informative to consider some recent advances in the structure and function of CMG helicase.

The CMG helicase is composed of the heterohexamer MCM2–7, which is the core motor of the helicase, along with Cdc45 and the GINS complex [87]. The MCM monomers each have a dumbbell shape which give the hexamer distinct N-terminal head and C-terminal tail domains, or tiers. The C-tier contains the AAA+ ATPase motors. The MCM2-7 complex is first assembled on double-stranded DNA as a head-to-head dimer. However, the active form of the helicase translocates in a 3′–5′ direction on the single-stranded leading strand template, N-tier first [88]. Thus, during origin activation, the lagging strand is extruded from the central channel [89] and the two active helicases pass each other on their respective leading strands [90]. The current structures of CMG support a modified steric exclusion model for DNA strand separation in which the parental duplex actually enters the central channel of the MCM hexamer [88] with the lagging strand exiting though a cleft in the top of the N-tier of CMG [91]. In the CMG complex, Cdc45 and GINS bind to the side of the MCM hexamer, contacting MCM2 and MCM5 where they have been proposed to act as a ‘lock’ to the ‘gate’ between these subunits. Opening of the interface between MCM2 and MCM5 may play an important role in the extrusion of the lagging strand [89].

Given that a pre-formed G4 will induce local strand separation, it seems likely that a lagging strand G4 may not actually enter the central channel but simply pass round the helicase. This can be considered analogous to the behaviour of the replisome when it encounters a bulky lesion (e.g., a tethered streptavidin molecule) on the lagging strand [92]. However, this ability of CMG to traverse lagging strand blocks is not simply a passive feature of the complex as, in the absence of the accessory protein MCM10, lagging strand blocks do stall the helicase leading to the proposal that MCM10 is required to drive a conformational change in CMG to make it permissive to a lagging strand impediment [93]. It will be thus be interesting to determine whether MCM10 is also required for CMG to traverse lagging strand G quadruplex structures. Once past the helicase, the G4 is likely to encounter the single-stranded binding protein RPA. Importantly, RPA has the capacity to destabilise and unfold G4s [54,94] meaning that the structure may be dissolved before it encounters DNA polymerase δ, the lagging strand polymerase.

What happens to a leading strand G4? There is no current evidence that the translocation of the CMG will unwind a G4 on its tracking strand, although it is plausible that the advancing helicase could disrupt some structures. If CMG does not disrupt the G4, is it feasible that the structure could pass through the channel of the MCM hexamer? When first assembled on DNA, before origin activation, the channel of the MCM double hexamer admits double-stranded DNA [95,96], which is approximately 2 nm (20 Å) in diameter. However, during origin activation the head-to-head MCM hexamers extrude the lagging strand as they are converted to the active CMG complex tracking on the leading strand template [89]. A high-resolution cryoEM structure of the *S. cerevisiae* CMG complex reveals that while the opening of channel into the N-tier of MCM remains about 2 nm, deeper into the channel constrictions are observed. The first are within the N-tier and are formed by intrusions from the OB domains of MCM2 and MCM7 [91]. In the C-tier, the constriction of the channel is more pronounced as the winged helix domain of MCM5 narrows it to c. 1 nm, which should be too small to accommodate dsDNA [91]. Nonetheless, CMG can translocate on a flush duplex suggesting that the internal channel can accommodate duplex DNA [97]. However, the diameter of G quadruplex structures is generally greater than duplex DNA at between 2.4 and 2.8 nm [98,99,100,101]. Thus, it would seem unlikely that most G4s would be able to pass through the channel of the active helicase, at least without some assistance. However, an intriguing genetic observation made in Marcel Tijsterman’s lab in the nematode worm *C. elegans* raises the possibility that preformed G4 may be able to survive the passage of CMG. In worms lacking Dog-1, the major G4 helicase in this species and orthologue of the human helicase FANCJ/BRIP1 [52], discussed further below, lineage tracing experiments reveal that deletions can be observed at the same G4 motif over multiple generations consistent with a G4 structure persisting through multiple cell cycles and generating deletions at each round of replication [26]. One explanation for this observation is that a G4 structure can persist through multiple rounds of cell division in the absence of Dog-1 without being unwound. How this might happen remains unclear. As noted above, direct passage of a leading strand G4 through the helicase seems unlikely. However, it is possible that structural rearrangements in the CMG helicase could allow it to tolerate tracking strand impediments. Such tolerance has been directly demonstrated for the SV40 replicative helicase, the large T antigen [102]: bulky translocation strand obstacles are traversed by large T, suggesting that the hexamer can transiently open. An analogous ability may exist in CMG, potentially through opening of the same interface, between MCM2 and MCM5, which allows lagging strand extrusion during origin firing [89]. Indeed, very recent work from Johannes Walter’s lab has demonstrated that the vertebrate CMG helicase is able to bypass a DNA-protein cross-link in the leading strand without dissociation [103].

### 5.4. Formation of G4s within the Replisome

As noted above, DNA unwinding significantly enhances the opportunity for secondary structure formation. Thus, the action of the CMG helicase could, per se, promote secondary structure formation in its wake. While RPA deposition on the lagging strand should counter the probability of secondary structure formation, there is currently no evidence that RPA binds to the leading strand template during otherwise unperturbed replication. Furthermore, despite the major recent advances in the structure of the budding yeast replisome, neither the precise path or extent of the leading strand between the helicase and polymerase is known [104]. However, it is likely that the length of the single-stranded DNA (ssDNA) tract between the helicase and polymerase varies as factors affecting progression of the DNA polymerase differ from those that affect the replicative helicase. In the extreme case of the leading strand polymerase being arrested, the helicase can continue to unwind the parental duplex for some considerable distance, a situation termed helicase-polymerase uncoupling. This leads to extensive RPA deposition [105,106]. Thus, it is reasonable to assume that continuous variations in the rate of DNA synthesis, caused by the nature of the sequence, nucleotide supply etc. will, in turn, lead to transient variations in the exposure of leading strand DNA. This could be sufficient to allow secondary structure formation within the replisome. Support for this idea comes again from experiments with the DT40 *BU-1* locus. Treating cells with chronic, low-dose hydroxyurea (HU), which depletes nucleotide pools, reducing the rate of DNA synthesis and promoting helicase-polymerase uncoupling, results in instability of *BU-1* expression. This is consistent with a model in which promoting the exposure of ssDNA between the helicase and polymerase promotes G4 structure formation and a more profound block to DNA synthesis than is induced by HU alone [58]. 

## 6. Countering G4 Formation during Replication

Factors that counter secondary structure formation can be considered in two groups, those that act indirectly to minimise structure formation by favouring B-form DNA and those that act directly to disassemble structures, pushing the DNA back to B-form. As discussed above, the formation of secondary structure is favoured by single-stranded regions of DNA. Therefore, factors that reduce the exposure of single-stranded DNA will be expected indirectly to reduce the probability that G4s will form, for example PrimPol-mediated repriming of DNA synthesis after a leading strand polymerase block [74]. In addition, proteins binding to ssDNA will reduce its ability to form secondary structures and further can drive folded G4s to unwind, as exemplified by RPA [54,94,107], REV1 [108] and, as discussed below, several of the ATP-dependent helicases proposed to possess ATP-independent modes of G4 unwinding, including BLM and DHX36 [109,110,111,112]. Working in parallel with these indirect or ATP-independent measures, a range of enzymatic activities have been implicated in actively disrupting G quadruplex structures. This includes specialised DNA polymerases, the role of which in DNA secondary structure metabolism we have reviewed recently [2], and specialised DNA helicases, which we consider next.

## 7. Helicases Involved in G4 Processing

Helicases are molecular motors that use ATP to alter nucleic acid structure. Unwinding of DNA and RNA double helices by helicases is necessary for virtually all cellular processes including replication, transcription, repair, recombination and chromosome segregation. Mutations in genes that code for helicases are linked to several human diseases, some with common characteristics like neurological problems, accelerated ageing and increased risk of cancer development [113,114]. We next summarise what is known about the key helicases that have been shown to play roles in G quadruplex metabolism. Importantly, all of these enzymes have significant roles in other pathways and, to date, no helicase solely dedicated to G4 unwinding has been identified (Table 1). However, we will highlight their additional roles in DNA repair and replication when a broader consideration of their functions helps illuminate the relevance of their contribution to unwinding secondary structures. In the absence of any clearly justifiable logic for grouping the helicases thus far implicated in G4 replication, we will order the discussion based on their structural features and organisation within the helicase superfamilies [115].

### 7.1. Superfamily 1: DNA2 and PIF1

#### 7.1.1. DNA2

DNA2 possesses ssDNA endonuclease activity on both 5′ and 3′ single-stranded overhangs [116] and a 5′-3′ helicase activity [117]. Human and yeast DNA2 can bind and unwind telomeric G4s in vitro, and can cleave such structures in the presence of RPA [118], to which the enzyme binds [119]. In vivo, reduction of DNA2 protein levels causes defects in telomere replication and increased levels of telomere aberrations in mouse cells [120]. However, the relative importance of the two catalytic activities for G4 resolution in vivo remains unclear.

#### 7.1.2. PIF1

The PIF1 family of 5′-3′ helicases are conserved from bacteria to humans [121] and all those studied so far are able to unwind G4 structures in vitro [122,123,124,125,126,127,128]. Although it is not a processive helicase [129], it is able to ‘trap’ a melted G4 in the unfolded state through binding single-stranded DNA [130]. In vivo, yeast and human PIF1 participate in G4 metabolism both of the nuclear (mainly telomeric) and mitochondrial genomes [131,132,133], with the latter predominating in the form of mitochondrial dysfunction in mutants of *PIF1* [134]. In budding and fission yeast G4 processing by Pif1 is important to preserve replication fork progression and supress chromosome breakage [39,55,56,124,135]. Pif1 associates with components of the replication fork [135] and its interaction with PCNA is needed for efficient replication through G4s [55]. There remains some uncertainty as to whether Pif1 operates predominantly on the leading or lagging strand as different systems and assays have produced conflicting results. The Nicolas group has clearly demonstrated that loss of Pif1 exacerbates genetic instability caused by leading strand template G4s [56]. Recently, an in vivo method to monitor fork progression between two points in the yeast genome [35] has revealed that overall fork delay at a G4 is increased in the absence of Pif1 when the G4 is on the lagging strand [55]. The immediate explanation for this discrepancy is unclear, and the two possibilities are naturally not mutually exclusive. However, these observations underscore potential differences between experimental approaches that monitor fork progression and those that record genetic changes, providing support for the idea that there is not necessarily a simple relationship between fork delay and the induction of genetic instability.

### 7.2. The Superfamily 2 RecQ-Like Helicases: BLM and WRN

RecQ helicases are a large protein family found in both prokaryotes and eukaryotes, all of which contain a core domain of approximately 450 amino acids that includes the conserved DExH box and that unwind with a 3′-5′ polarity. Human cells have five RecQ helicases, two of which have prominent roles in G4 metabolism, BLM, mutated in Bloom’s syndrome [136] and WRN, mutated in Werner’s syndrome [137]. Mechanistically, ATP-dependent G4 unwinding by RecQ helicases is well characterised and involves DNA binding to the RecQ C-terminal domain (RQC), which is responsible for recognition of several DNA structures [138]. Recently, a structure of a bacterial RecQ bound to a resolved G4 provides an important clue to the mechanism of this enzyme that may be more generally applicable to the entire RecQ family. A guanine specific pocket (GSP) sequesters dG from the end of the G4 in a manner that would not be compatible with its participation in a folded G4. This suggests that the enzyme binds to a folded G4 and flips out a guanine at the base of the structure. If performed sequentially, this action would destabilise the structure [139].

#### 7.2.1. BLM

BLM was one of the first human helicases reported to resolve G4s [140]. In vitro, BLM preferentially binds to and unwinds multi-stranded intermolecular G4s over duplex DNA and branched substrates like double Holliday junctions [138,141,142]. BLM requires a short 3′ single-stranded overhang in order to load onto DNA for G4 unwinding [140] and the presence of a G4 within 6 nt of the end of this tail inhibits its unwinding activity [143]. BLM is less active on more physiologically relevant intramolecular G4s [144] and its activity is inhibited by G4 binding ligands [145], which trap the enzyme in a state in which it hydrolyses ATP but is unable to continue unwinding or dissociate [141]. More recent single-molecule experiments have provided further insight into the mechanism by which BLM destabilises G4s. The ATP-dependent unwinding of intramolecular G4s arises by the enzyme reeling in single-stranded DNA adjacent to the structure [146,147]. However, when this mechanism is unsuccessful, BLM dissociates from the DNA, in contrast to PIF1, which remains bound and makes repeated attempts at unwinding [147]. Somewhat surprisingly, the binding of BLM to ssDNA adjacent to a G4 can per se destabilise the G4 in the absence of ATP [109]. This passive G4 resolution effect is dependent on the underlying stability of the G4 and is observed with WRN (see below) but not other RecQ helicases [109], and is reminiscent of G4 destabilisation by other proteins that bind ssDNA such as RPA [54] and REV1 [108]. Interestingly, RPA also directly facilitates the helicase activity of BLM [148] and, recently, the interaction between both BLM and WRN and RPA has been shown to be promoted by the E3 ubiquitin ligase HERC2 [149], showing that additional factors are likely to be necessary to promote efficient deployment of these helicases at G4s in vivo.

In vivo experiments also support BLM playing an important role in G4 processing. The *C. elegans* BLM homolog HIM-6 limits deletions of G-tracts in the absence of the FANCJ homolog *dog-1* [150]. A second line of in vivo evidence for BLM playing a role in countering G4 formation comes from a curiously consistent feature of cells lacking BLM and WRN helicases: dysregulated expression of genes harbouring G4s motifs in the vicinity of their transcription start sites (TSS) [57,151,152,153,154]. The explanation for this phenomenon is likely to be multifactorial. For those genes with G4s in the promoter regions, the altered gene expression may relate to a direct effect of persistent G4 formation on transcription factor binding or nucleosome positioning. However, putative G4s are also enriched downstream of the TSS in dysregulated genes and may alter expression through replication-dependent epigenetic instability [57].

Telomeres contain the highest density of potential G4-forming sequence in the genome due the nature of the telomeric repeat TTAGGG, which forms the template for lagging strand synthesis. It is not surprising, therefore, that helicases with potential to unwind G4s play an important role in telomere maintenance, BLM included. BLM interacts with POT1, an OB fold-containing telomeric single-stranded binding protein [155], and mouse cells deficient for BLM are impaired in correct replication of G4-forming telomeric sequences, resulting in increased telomeric instability [32,156].

#### 7.2.2. WRN

In addition to 3′-5′ helicase activity, WRN possesses exonuclease activities and single-strand annealing activities [157,158]. WRN can resolve a variety of secondary structures including G4s and triplexes in vitro [142,159,160] and, like BLM, it has ATP-dependent and independent modes of unwinding, requiring a short 3′ ssDNA tail and is stimulated by RPA [158,161,162]. WRN and BLM also physically interact and it has been suggested that the two helicases cooperate in vivo [163]. However, WRN and BLM also exhibit considerable functional redundancy in maintaining G4-dependent epigenetic and transcriptional stability in DT40 cells [57].

Like BLM, WRN plays an important role at telomeres. In vitro, WRN interacts with pol δ and this interaction enables pol δ to replicate through G4s, suggesting that WRN could play a role in telomeric lagging strand replication [164]. Supporting this, in shuttle vector experiments, WRN is essential for the replication of a (TTAGGG)_6_ telomeric repeat, its absence increasing the frequency of large deletions and rearrangements [165], which may explain why WRN-deficient cells exhibit loss of the G-rich telomere lagging strand [166]. Further control of WRN at telomeres is likely to come from specific interactions with telomeric proteins including POT1 [32,155] and the shelterin complex proteins, which regulate WRN’s catalytic functions on telomeric D-loops [167]. The role of WRN at the telomeres and its link to preventing premature ageing is underscored by the observation that telomerase-deficient mice with short telomeres that also lack WRN (Terc^−/−^ Wrn^−/−^) exhibit dysfunctional telomeres and chromosome instability [168,169,170].

### 7.3. The Superfamily 2 Fe-S Helicases: RTEL1, DDX11, FANCJ and XPD

Fe-S helicases are so named because they contain iron-sulphur clusters, ancient protein co-factors which play an essential role in protein folding and domain stabilisation [171] and found in many DNA replication and repair proteins [172]. All four of these helicases have the ability to unwind G4s in vitro but, as discussed below, the link between G4 processing and the human syndromes associated with the mutation of XPD remain unclear.

#### 7.3.1. FANCJ (Also Known as BRIP1 and BACH1)

FANCJ is an ATP-dependent 5′-3′ helicase mutated in a rare subtype of Fanconi Anaemia [173,174]. It is able to unwind duplex DNA, branched DNA structures and D-loops [175], as well as several forms of G4 on which it exhibits greater activity than it does on duplex DNA [27,176]. In addition to its helicase core that binds and translocates along ssDNA, FANCJ also possesses a G4 recognition site [177]. As with other G4 disrupting helicases, it requires the presence of a short ssDNA tail (in this case 5′ of the structure) for optimal G4 unwinding activity and is stimulated by RPA [175,176].

FANCJ has been detected associating with the elongating replication fork [178,179] and with telomeres [180]. As for PIF1, there is evidence for FANCJ operating on both leading and lagging strands. Worms deficient in the FANCJ orthologue *dog-1* exhibit deletions in G4 motifs that have the potential to form G4 structures during lagging strand replication [38,52,150]. However, in chicken DT40 cells, FANCJ can clearly suppress the formation of G4 structures on the leading strand template [57]. This ability of FANCJ to manage G4s at the fork plays an important role in the ability of cells to maintain chromatin states through replication [57,181].

Interestingly, the function of FANCJ in resolving G4s appears to be independent of much of the canonical FA pathway. Deficiency of FANCA or FANCD2 in human cells does not lead to sensitivity to the G4 ligand telomestatin [27] and the G4-dependent instability of expression of the *BU-1* locus of DT40 seen in FANCJ-deficient cells is not observed in a FANCC mutant [57]. Likewise, the persistent fork stalling that occurs during replication of a G4-containing plasmid in a FANCJ-depleted *Xenopus* egg extract is not seen following depletion of FANCD2 [182]. Nonetheless, the double mutant of FANCJ and FANCD2 in *C. elegans* shows a higher mutation rate at G4 motifs compared to the single FANCJ mutant, suggesting these proteins may play distinct roles in promoting G4 resolution [183].

#### 7.3.2. DDX11 (Also Known as CHLR1)

DDX11 is an essential gene in mice [184]. In humans, mutations in DDX11 cause Warsaw Breakage Syndrome, an extremely rare inherited disorder characterised by microcephaly, growth retardation, cochlear abnormalities and abnormal skin pigmentation [185]. DDX11 is capable of ATP-dependent unwinding of G4s [186,187], as well as other DNA structures such as triplex DNA [188] and D-loops [187]. Interestingly, and despite the homology between the helicase core of DDX11 and FANCJ, the two enzymes exhibit distinct preferences in terms of the types of G4 they are capable of unwinding in vitro. FANCJ proficiently unwinds a unimolecular G4, while the same structure defeats DDX11 [186]. However, DDX11 is capable of unwinding intermolecular G4s formed between two DNA strands [186,187], which is of potential, but unproven relevance in the origin of the most prominent phenotype resulting from its inactivation, defects in sister chromatid cohesion [184,189,190,191,192]. DDX11 interacts with Timeless [193,194], a core component of the eukaryotic replisome, potentially placing it in an ideal position to ‘sweep’ G4s that inhibit progression of the replicative polymerases.

#### 7.3.3. RTEL1

Regulator of telomere elongation helicase 1 (RTEL1), as its name suggests, plays a prominent role in telomere maintenance. Purified RTEL1 is a 5′-3′ helicase able to catalyse ATP-dependent unwinding of an intramolecular G4 formed by the human telomeric repeat, in and ATP-dependent manner, in vitro. In the absence of RTEL1, telomeric D-loops (T-loops) are not correctly resolved during replication, resulting in loss of telomeric sequence [156]. Furthermore, RTEL1 also suppresses telomeric instability due to G4s as telomere fragility in the absence of RTEL1 is vastly increased if BLM is also removed or G4s are stabilised [156]. Since T-loops are suggested to protect telomeres from de-stabilizing events [195], the biochemical activity of RTEL1 is consistent with a predicted function of this helicase to facilitate the replication of the 3′ G-rich overhang [196]. RTEL1 deficiency is embryonically lethal in mice [53], caused by compromised telomere elongation in ES cells [197].

RTEL1 also plays a role in replication of non-telomeric DNA. It binds to the replisome via its interaction with PCNA. Loss of this interaction leads to reduced replication fork velocity and increased fork instability [198].

#### 7.3.4. XPD/ERCC2

XPD is one of the helicases of the TFIIH complex, implicated in basal transcription and nucleotide excision repair (NER) [199]. XPD binds and unwinds G4 structures in vitro, while XPB (the other TFIIH helicase) only binds G4 DNA [200]. The same study also found that XPD and XPB binding sites overlap with G4 motifs, which suggests that TFIIH is recruited to G4 structures; moreover, XPD and XPB binding sites are enriched in the vicinity of TSS of highly transcribed genes. However, the in vivo significance of XPD in replicating G4 sequences and its relationship to the other crucial functions of this helicase remains to be dissected.

### 7.4. The Superfamily 2 DEAD-Box Helicases: DHX36 (Also Known as RHAU/G4-Resolvase 1) and DHX9

#### 7.4.1. DHX36

DHX36 was first identified as an RNA helicase recruited to an AU-rich element in the urokinase plasminogen activator messenger RNA (mRNA), hence its name RHAU. In this context, it acts to facilitate mRNA deadenylation and mRNA decay by the exosome [201]. It was subsequently identified as responsible for the dominant G4 unwinding activity in HeLa cell lysate [202,203]. It is an essential gene being necessary for normal embryogenesis [204,205].

DHX36 has an extremely high affinity for DNA and RNA G4s [206,207]. This is mediated in part by a 13 amino acid N-terminal motif, the DHX36 specific motif (DSM) [208], and in part by contacts made by the C-terminal OB fold domain [110]. The enzyme exhibits robust ATP-dependent resolving activity on both RNA and DNA G4s, including tetramolecular and unimolecular structures [202,207,209,210], but is specific for parallel G4s [211,212,213].

Recent structural, biochemical and biophysical studies have shed light into an ATP-independent mode of G4 unwinding by DHX36 [110,112,211,213]. A crystal structure of *Bos taurus* DHX36 bound to the *MYC* promoter G4 confirms a mode of binding similar to many G4 ligands in which the core of the DSM creates a flat, non-polar surface which interacts with the top quartet of the G4 [110]. The energy that comes from binding of the nucleic acid is transduced by the enzyme into a directed pulling force arising from rotation of the C-terminal domain and opening of the helicase core. This results in the parallel G4 substrate being unwound sequentially by one nucleotide at a time without the need for ATP hydrolysis, providing further evidence for the importance of ATP-independent modes of G4 destabilisation. Interestingly, ATP hydrolysis is needed for release of the substrate and when the enzyme is bound to ADP or the non-hydrolysable analogue AMP-PNP it actually stabilises G4 structures against mechanical unfolding [214]. This demonstrates the principle that a helicase can exhibit opposing actions in terms of G4 unwinding and stabilisation, a point discussed further below.

Similarly, the crystal structure of *Drosophila melanogaster* DHX36 bound to the *MYC* G4 showed that DmDHX36 has a positively charged pocket that destabilizes the G4, and also displays a passive, ATP-independent manner of G4 destabilisation upon binding to the DmDHX36 pocket [111]. However, to complete unfolding of the G4 DNA, an ATP hydrolysis translocation step is needed. The authors propose a ‘passive-active’ G4 unwinding mechanism, combining ATP-independent partial G4 destabilisation with ATP-hydrolysis driven unfolding [111].

#### 7.4.2. DHX9 (Also Known as Nuclear DNA helicase II/RNA Helicase A)

DHX9 is emerging as a potentially important DNA G4 processing factor. It is able to unwind G4s in vitro [215] and interacts with PCNA [216], topoisomerase IIα [217] and WRN [218]. Consistent with it playing a role in DNA replication, human fibroblasts depleted of DHX9 exhibit significant replication defects and p53-dependent senescence [219].

## 8. Coda: Principles Governing the Recruitment of G4 Helicases

The prevalence of potential structure-forming sequences suggests that DNA itself could pose one of the most significant barriers to replication with structural impediments being at least as frequent as those created by spontaneous DNA damage. This is supported by recent experimental data, which also suggest that secondary structure formation must be actively managed to ensure timely replication with the preservation of genetic and epigenetic stability. In this final section, we consider how the mechanisms and enzymes we have discussed above are selected and coordinated to ensure secondary structures are eliminated in order to allow the completion of DNA synthesis.

### 8.1. Selection 

By analogy with the mechanisms that ensure timely replication of DNA damage [220], it is likely that a combination of context and the nature of the impediment contribute to determining which mechanism is selected. In terms of how specialised helicases are recruited to a G4, this problem can be distilled to three factors: G4 conformation, the availability of 5′ and/or 3′ ssDNA and specific protein–protein interactions.

G4s can adopt a wide range of conformations, with many complex G4 sequences exhibiting significant conformational polymorphism. The idea that G4 helicases may exhibit some selectivity for specific G4 arrangements emerges from the comparison of the in vitro behaviour of DHX36, BLM and FANCJ. DHX36 exhibits a significant preference for unwinding parallel G4s, while BLM is non-selective [213]. Likewise, although DDX11 and FANCJ belong to the same family they exhibit different abilities to unwind a unimolecular parallel G4 [186]. However, an important unresolved issue is the extent to which this apparent selectivity for specific structures occurs in vivo.

It will also be evident from the preceding discussion that G4 helicases generally require a ssDNA ‘landing pad’ at either the 5′ or 3′ end of the G4. This, too, will contribute to the selection of helicases with appropriate polarity. The circumstances under which such ssDNA tracts are generated around G4s are poorly understood. For example, during replication, repriming after a leading strand stall will leave a 5′ ssDNA adjacent to the G4, which would provide a platform for the recruitment of a helicase such as FANCJ [57]. Other situations, e.g., a lagging strand stall may favour the recruitment of helicases with 3′ polarity, which may to some extent explain the important roles played by BLM and WRN at telomeres, but it is clear that such any leading and lagging strand specialisation is not likely to be determined by where ssDNA is formed round a G4. Whether a specific helicase recruited to the ssDNA near a G4 is then able to unwind the structure is then also likely to be determined by a combination of the nature of the G4, the ability of the helicase to unwind that structure and its processivity. This can be illustrated by the in vitro behaviour of BLM and PIF1, both of which can unwind a broad range of G4s but exhibit different abilities to remain bound in the event of a failed attempt [147]. We have noted numerous specific protein–protein interactions in the above discussion and these are also likely to play an important role determining which helicases are recruited to the vicinity of a G4 in different contexts e.g., to the leading or lagging strand or to telomeres. Similar to the recruitment of translesion polymerases to sites of lesion bypass [221], there may be a ‘two-step’ process in which the helicases are concentrated near where they are likely to be needed and then separate interactions, including those with the G4 and ssDNA nucleic acid structures promoting the actual unwinding step.

All that said, the idea that helicases simply act to remove secondary structures is likely to be an oversimplification. Drawing parallels with RNA metabolism, in which helicases often simply remodel or chaperone secondary structure during, for instance, splicing or translation [222], it is possible that similar transitions are promoted in DNA secondary structures, for instance to render them more or less amenable to unwinding, as illustrated by the behaviour of DHX36 [214]. Thus, in some cases, a helicase may actually promote, rather than alleviate the potential replication impediment caused by a secondary structure.

### 8.2. Collaboration

It is likely that the multiple available mechanisms for G4 unwinding in vertebrates exhibit significant redundancy to ensure the cell mounts a robust response to the challenges imposed by secondary structure during replication of large and complex genomes. Some preliminary evidence for this comes from our studies of the stability gene expression in the *BU-1* locus and across the genome in DT40 cells, which suggested that BLM and WRN exhibit considerable functional redundancy in terms of G4 processing [57]. The same study also provided evidence that helicases can collaborate and speculated that 5′-3′ and 3′-5′ helicases could operate together to unwind a G4 from both ends, or that a 5′-3′ helicase could collaborate with specialised DNA polymerases, like REV1, acting from the stalled 3′ primer terminus [57]. In additional to this simultaneous cooperation, it is also possible that helicases may act sequentially with a first enzyme remodelling a G4 into a form that is more amenable to unwinding by a second enzyme.

Now that the formation of G4s in vivo is more widely accepted and we have a catalogue of factors that counter G4 formation, albeit an incomplete one, a significant challenge for the coming years is to understand how the extensive roster of mechanisms discussed above are recruited, coordinated and regulated.

## Figures and Tables

**Table 1:** Summary of the G4 processing helicases discussed in this review.

Superfamily	Subfamily	Name	Substrate *	Polarity	Other Functions ¶	Interactions #	Human Syndrome
SF1		PIF1	DNA (parallel and	5′–3′	Maintenance of	PCNA	L319P mutation linked to
			antiparallel, intramolecular ¶ and tetramolecular G4)		mitochondrial genome, DSB ¶ repair		familial breast cancer
		DNA2	DNA (telomeric G4)	5′–3′	Okazaki fragment	RPA	
					maturation, ICL repair		
SF2	Fe-S	FANCJ	DNA (parallel, intramolecular and tetramolecular G4, D-	5′–3′	ICL repair, checkpoint control, replication stress	RPA, WRN, BLM, REV1	Fanconi Anaemia (FA), breast cancer
			loops)		response		
		DDX11	DNA (antiparallel,	5′–3′	Sister chromatid cohesion,	PCNA, RPA, FEN1,	Warsaw Breakage Syndrome (WABS)
			intramolecular G4, triplex, 5′		post-replicative repair	Ctf18-RFC,	
			flap, D-loop)			Timeless-Tipin	
		RTEL1	DNA (telomeric G4)	5′–3′	ICL repair, replication stress	POT1, PCNA	Dyskeratosis congenita
					response		
		XPD	DNA (parallel tetramolecular	5′–3′	NER, basal transcription		Xeroderma Pigmentosum,
			G4)				Cockayne syndrome, Trichothiodystrophy (TTD), ¶ Cerebro-oculo-facio-skeletal syndrome (COFS)
	RecQ	WRN	DNA (intermolecular parallel	3′–5′	Fork protection and	RPA, pol δ, FANCJ,	Werner syndrome
			G4, triplex, Holliday Junction)		remodelling, replication	POT1, BLM	
					stress response, checkpoint		
					control, DSB and ICL repair		
		BLM	DNA (intermolecular and	3′–5′	Fork protection and	RPA, pol δ, FANCJ,	Bloom syndrome
			tetramolecular parallel G4,		remodelling, DSB and ICL	POT1, WRN	
			triplex, Holliday Junction)		repair		
	DEAH	DHX9	RNA and DNA (intramolecular and tetramolecular parallel	3′–5′	Replication stress response, transcription regulation	PCNA, TOP2α, WRN	
			G4, R-loop)				
		RHAU/DHX36	RNA and DNA (intramolecular	3′–5′	Translation regulation, RNA		
			and tetramolecular parallel ζ G4, R-loop)		decay, telomere length ζ regulation		

* substrates other than B-DNA; # interactions linked to activity on structured DNA; ¶ functions other than secondary structure processing.
